# Exploring Needle-Like Zinc Oxide Nanostructures for Improving Dental Resin Sealers: Design and Evaluation of Antibacterial, Physical and Chemical Properties

**DOI:** 10.3390/polym12040789

**Published:** 2020-04-02

**Authors:** Fabrício M Collares, Isadora M Garcia, Mariana Klein, Clarissa F Parolo, Felipe Antonio L Sánchez, Antônio Takimi, Carlos P Bergmann, Susana Maria W Samuel, Mary Anne Melo, Vicente CB Leitune

**Affiliations:** 1 Dental Materials Laboratory, School of Dentistry, Federal University of Rio Grande do Sul, Rua Ramiro Barcelos, 2492, Rio Branco, Porto Alegre 90035-003, RS, Brazil; isadora.garcia@ufrgs.br (I.M.G.); mariana.klein@ufrgs.br (M.K.); susana.samuel@ufrgs.br (S.M.W.S.); vicente.leitune@ufrgs.br (V.C.B.L.); 2 Biochemistry and Microbiology Laboratory, School of Dentistry, Federal University of Rio Grande do Sul, Rua Ramiro Barcelos, 2492, Rio Branco, Porto Alegre 90035-003, RS, Brazil; clarissa.parolo@ufrgs.br; 3 Laboratory of Ceramic Materials, Federal University of Rio Grande do Sul, Av. Osvaldo Aranha, 99, Porto Alegre 90035-190, RS, Brazil; felipe.sanchez@ufrgs.br (F.A.L.S.); carlos.bergmann@ufrgs.br (C.P.B.); 4 Laboratory for Electrochemical Processes and Corrosion, Engineering School, Federal University of Rio Grande do Sul, Bento Gonçalves, 9500, Prédio 43427, Sala 216, Porto Alegre 91501-970, RS, Brazil; antonio.takimi@gmail.com; 5 Ph.D. Program in Biomedical Sciences, University of Maryland School of Dentistry, Baltimore, MD 21201, USA; 6 Operative Dentistry Division, General Dentistry Department University of Maryland School of Dentistry, Baltimore, MD 21201, USA

**Keywords:** methacrylate-based materials, zinc oxide, Biopolymers, root canal filling materials, Antimicrobial agents, dental materials

## Abstract

This study aimed to evaluate the effect of needle-like zinc oxide nanostructures (ZnO-NN) on the physical, chemical, and antibacterial properties of experimental methacrylate-based dental sealers. ZnO-NN was synthesized and characterized. ZnO-NN was added to a co-monomer blend at 20, 30, and 40 wt.%. One group without ZnO-NN was used as a control. The dental resin sealers were evaluated for their flow, film thickness, water sorption, solubility, radiopacity, degree of conversion (DC), dental-sealer interface characterization via micro-Raman, and antibacterial activity. ZnO-NN presented a mean needle diameter of 40 nm and 16 m^2^/g of surface area. There was no difference among groups containing ZnO-NN regarding their flow. The ZnO-NN addition significantly increased the film thickness. Water sorption and solubility tests showed no difference among groups. The radiopacity increased, and DC decreased with higher concentrations of ZnO-NN. Micro-Raman suggested that ZnO-NN was in close contact with root canal dentin. Overall, the incorporation of ZnO-NN provided an antibacterial effect against *Enterococcus faecalis* without a significant detrimental impact on the physical and chemical functionality of the material. The use of ZnO-NN as an inorganic filler is a potential application within dental materials intended for root canal treatment.

## 1. Introduction

Nanomaterials have received widespread attention for dental applications partly because they exhibit improved or new functionality in comparison to their microsized counterparts [1,2,3]. In dentistry, macro and micro-sized zinc oxide (ZnO) are well-stabilized inorganic fillers and have been tested in dental materials. Sealers and cement (zinc oxide-eugenol and zinc phosphate cement) [4,5], composites [6], and adhesive resins [7] are among the materials in which ZnO has been explored.

As biointeractive fillers, nanosized ZnO particles can enable mineral growth via bioactivity and negatively affect metalloproteinases and bacteria growth [8,9,10]. Particles with nanometer-sizes present a high surface area and increased surface reactivity due to a higher percentage of atoms on the material’s surface [11]. Therefore, their biological properties may be intrinsically associated with their low dimensionality and higher reactivity [1,12]. Moreover, the decreased size of fillers can lead to different physical and chemical properties of materials [7].

Over the past decade, the synthesis, characterization, and potential biomedical applications of one-dimensional nanostructure ZnO have been studied [13,14]. A large variety of ZnO nanostructures, such as nanoamorphous particles, nanowires, nanotubes, and nanofibers, has been prepared [15]. Also, some novel ZnO with complex architectures, such as nanocombs, nanosprings, nanoneedles, nanopropellers, tetrapods- and flower/urchin-like nanostructures have been reported in the literature up to date [16]. The variability on the morphology of ZnO has been explored as a pathway for the development of new properties, investigations of cell adhesion, and promising improved antibacterial effect [17,18].

The antibacterial performance of ZnO nanostructures depends on their size and shape, surface-to-volume ratio, and the number of oxygen vacancy sites [1,19]. ZnO nanostructures hamper the growth of *Enterococcus faecalis*, an essential bacterium in the post-infection of root canal treated teeth, and failure of treatment [20]. This antibacterial activity occurs via membrane disorganization and gene downregulation promoted by ZnO [18,21]. These outcomes are exciting and lead to the questioning of whether ZnO nanostructures could be used as bioactive inorganic fillers in dental sealers for root canal treatment. However, the maintenance of a range of functionalities of dental sealers during the root canal obturation is essential to attain the desired treatment outcome. Ideally, dental sealers should be capable of (1) penetrating and sealing the dentinal tubules; (2) binding intimately to both the organic and inorganic phases of dentin; (3) decreasing microorganisms viability and neutralizing their products; (4) predictably inducing a regenerative response, and (5) strengthening the root complex system [22,23]. 

**Figure 1:**
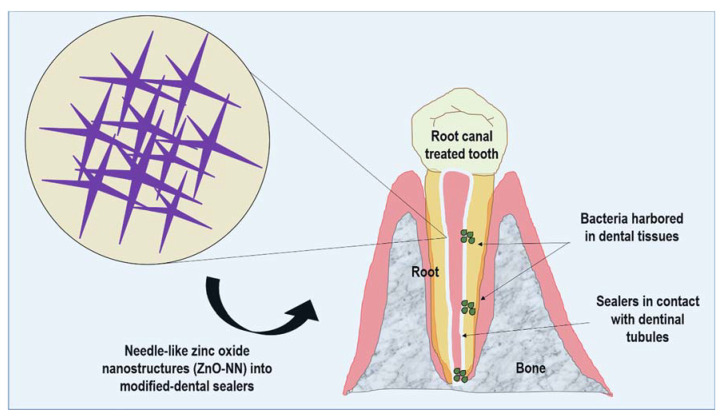
Schematic representation of the proposed approach of needle-like zinc oxide nanostructures (ZnO-NN) for improving dental resin sealers.

Nanostructured needle-like zinc oxide (ZnO-NN) presents extensions originating from one nucleation core with distinct directions within a three-dimensional space [24]. This structure results in a uniform dispersion and homogeneous stress distribution into the materials, which may reflect in their reinforcement [25]. Subsequently, the prospects of using nanostructures of ZnO with needle-like shapes may be an exciting step to achieve better dental sealers for endodontics (Figure 1).

Based on the above consideration, this study reports the synthesis of needle-like zinc oxide nanostructures (ZnO-NN) particles and their incorporation into an experimental methacrylate-based dental sealer for root canal treatment for the first time. This study aimed to assist the investigators in determining whether a candidate ZnO nanostructure has scientific merit to justify further investigations and applicability. For this purpose, we evaluated the dental sealers for the following physical, chemical, or biological properties: flow, film thickness, water sorption, and solubility, radiopacity, degree of conversion, root-sealer interface, and antibacterial effects against *E. faecalis*.

## 2. Materials and Methods

### 2.1. Synthesis of Needle-Like Zinc Oxide Nanostructures (ZnO-NN)

Needle-like zinc oxide nanostructures were prepared by a simple thermal evaporation method, according to a previous study [26]. Briefly, ZnO powder mixed with graphite (molar ratio 1:1) was placed at the closed end of the quartz tube and then inserted into a horizontal tube furnace heated to 1100 °C. The temperature gradient at the location between the source material and the open end of the quartz tube was approximately 600 °C. After 30 min of evaporation, the quartz tube was removed from the furnace and left to cool down at room temperature. White color power was observed in the inner walls of the quartz tube. Particle size and particle shape were analyzed by scanning electron microscopy (TM3000 model, Hitachi High-Technologies, Naha-shi, Okinawa, Japan). The Brunauer–Emmett–Teller (BET) method was used to analyze the specific surface area via an automated gas sorption system (Quantachrome NOVA1000 Autosorb, Boynton Beach, FL, USA).

### 2.2. Resin Sealers Formulation

The experimental dental sealers were formulated using urethane dimethacrylate (UDMA), glycerol-1,3-dimethacrylate (GDMA), ethoxylated bisphenol A glycol dimethacrylate (BISEMA), camphorquinone (CQ), N, N-dihydroxy ethyl-para–toluidine (DHEPT) and benzoyl peroxide (BP). These materials were used without further processing. Table 1 describes the chemical composition of the formulations assessed in this study. ZnO-NN was added at three different weight ratios (20, 30, or 40 wt.%) over the total quantity of base resin (monomers and photoinitiator system). One group without the addition of ZnO-NN was used as a control. The mixture (base resin/ZnO-NN) was hand-mixed for 60 s, sonicated for 90 s, and hand-mixed again for 60 s. A light-emitting diode activation unit (1200 mW cm^−2^, Radii; SDI, Bayswater, Australia) was used to photoactivate the sealers along with all study.

### 2.3. Flow

The flow test was conducted in accordance with ISO 6876:2012 [27]. A total of 0.5 ml of each experimental sealer was placed on a glass plate (40 × 40 × 5 mm) with a graduated syringe. At 180 ± 5 s after the start of mixing, another plate with a mass of 20 ± 2 g and a load of 100 g was applied on top of the material. Ten minutes after the start of mixing, the weight was removed, and the major and minor diameters of the compressed material were measured using a digital caliper. If both measurements were consistent to within 1 mm, the results were recorded. If the major and minor diameter discs were not uniformly circular or did not match within 1 mm, the test was repeated. For each experimental group, the test was conducted three times, and the mean value was taken.

### 2.4. Film Thickness

The film thickness evaluation was conducted following the ISO 6876:2012 [27]. Two glass plates (5 mm thick and 40 mm side) were placed together, and their combined thickness was measured. A drop of 0.5 ml of the experimental sealer was placed at the center of one of the plates. Another plate was placed on top of the material. At 180 ± 5 s after the start of mixing, a load of 150 N was applied vertically onto the top glass plate. Ten minutes after the mixing, the thickness of the two glass plates and the interposed sealer film was measured using a digital caliper. The difference in the thickness of the two glass plates, with and without a sealer, was the film thickness of the experimental sealer material. The mean value of three measurements for each sealer was taken as the film thickness of the material.

### 2.5. Water Sorption and Solubility

Water sorption and solubility tests were performed according to ISO 4049:2009 [28], except for the dimensions of the specimen (10.0 ± 0.1 mm diameter, 1.0 ± 0.1mm thickness). The samples (n = 5) were stored in a dissector at 37 °C containing fresh silica gel dried and maintained at 37 °C. Each sample was weighed to an accuracy of 0.01 mg at repeated intervals of 24 h in an analytical balance (AUW220D, Shimadzu, Tokyo, Japan) until a constant mass (*m*_1_) was obtained (i.e., until the mass loss of each specimen was not more than 0.1 mg in 24 h). After final drying, two measurements of the diameter, at right angles to each other, were taken with a digital caliper, and the mean diameter was calculated. The thickness of the specimen was measured at the center of the sample and at four equally spaced points on the circumference. The area was calculated, in mm^2^, from the mean diameter, and then, using the mean thickness, the volume was calculated in mm^3^. 

The samples were stored in water at 37 °C for seven days; the volume of water for immersion was 10 mL *per* sample. Afterward, the samples were removed from the liquids, which were stored in a light-free container. Each specimen was weighed after being dried slightly to blot away the surface water, and the weight was obtained (*m*_2_), i.e., the mass of the hydrated samples. After weighing, the samples were returned to the first desiccator. The cycle was repeated until the constant weight was recorded as *m*_3_. Water sorption (WS) was calculated according to Equation (1), and the solubility (SL) according to Equation (2) [29].

(1) $$\mathrm{WS}=\frac{\left(m_{2}-m_{3}\right)}{V}$$

(2) $$\mathrm{SL}=\frac{\left(m_{1}-m_{3}\right)}{V}$$

### 2.6. Radiopacity

The radiopacity of the experimental sealers was performed according to ISO 6876:2012 [27]. Five samples per group were produced with 10.0 mm (±0.1 mm) in diameter and 1.0 mm (±0.1 mm) thick. Radiographic images were obtained using a phosphor plate digital system (VistaScan; Dürr Dental GmbH, Bietigheim-Bissingen, Germany) at 70 kV and 8 mA, with 0.4 s of exposure time and a focus-film distance of 400 mm. For each film, one specimen from each concentration was positioned for a total of four samples per film. An aluminum step-wedge was exposed simultaneously with the samples in all images. The aluminum step-wedge thickness ranged from 0.5 to 5.0 mm in increments of 0.5 mm [30]. The aluminum alloy used was Al 99.12, Fe 0.47, Mg 0.41, and with <0.1 of Cu (wt.%). The images were saved in TIFF format and analyzed using Photoshop software (Adobe Systems Incorporated, San Jose, CA, USA). The means and standard deviations of the grey levels (pixel density) of the aluminum step-wedge and the samples were obtained in a standardized area of 1.5 mm^2^.

### 2.7. Degree of Conversion

The degree of conversion (DC) was measured by Fourier Transform Infrared Spectroscopy (FTIR) with a spectrometer (Vertex 70, Bruker Optics, Ettinger, Germany) equipped with an attenuated total reflectance device, composed of a horizontal diamond crystal with a mirror angle of 45 ° [31]. A light emitting diode curing unit (Radii; SDI, Bayswater, Australia) with an irradiance of 1200 mW/cm^2^ was fixed on support to standardize the distance between the tip and the sample at 5 mm. The sample (uncured composite) was directly dispensed (3 µL) on the top of the diamond crystal and photoactivated for 60 s (n = 3). Data were evaluated with the Opus software (Bruker Optics, Ettlingen, Germany), with Blackman–Harris 3-Term apodization in a range of 4000 to 400 cm^−1^ and a resolution of 4 cm^−1^. The DC was calculated based on the intensity corresponding to the stretching of the carbon–carbon double bond (peak height) at 1635 cm^−1^, and a symmetric ring stretching at 1610 cm^−1^ of non-polymerized and polymerized samples [32]. The analyses were performed with the same samples after seven and 14 days stored at 37 °C.

### 2.8. Interface Characterization by Micro-Raman 

Four lower incisor human teeth were cleaned of organic debris and stored in distilled water at 4 °C. The roots were sectioned below the enamel cement junction to obtain roots with a final length of 15 mm. The root canals were chemical-mechanically prepared with first series Kerr files until 40 files, in an ascending sequence, using a step-back technique, associated with sodium hypochlorite 1% irrigation. The canals were prepared 1 mm below the final strength. After the chemical mechanical preparation, the canals were irrigated with 3 mL EDTA for 1 minute and then washed with 3 mL of sodium hypochlorite and 3 mL of distilled water. The canals were dried with absorbent paper cones. The roots were then randomly divided and filled with the experimental sealer and gutta-percha cones as core material. The sealer was photoactivated for 40 s from the top of the root canal cervical portion and stored at 37 ± 1 °C for seven days. 

After this period, the roots were sectioned at low speed under constant irrigation (Low-Speed Saw, Buehler, Lake Bluff, IL, USA) perpendicular to the long axis of the root. A scan in mapping mode by Raman vibrational spectroscopy (micro Raman) was performed in line with 50 µm in each slice, including sealer and dentin, so it was possible to characterize the interface and analyzed the sealer penetration into dentin. Micro-Raman spectroscopy was performed using a SENTERRA Raman Microscope (Bruker Optics, Ettlingen, Germany). It was used 785 nm laser for 10 s with five co-additions, totaling 60 s with 100 mW of laser power, resolution of 3–5 cm^−1^, and spectra were analyzed between 80 and 1525 cm^−1^. 

### 2.9. Antibacterial Activity

To evaluate the antibacterial effect of the dental sealers against *Enterococcus faecalis,* a collection strain of this bacterium (ATCC 29212; American Type Culture Collection, Rockville, MD, USA) was used in this study. Bacteria from frozen stock cultures were grown aerobically in Brain Heart Infusion (BHI) broth (HiMedia Laboratories Pvt.Ltd, Mumbai, India) at 37 °C. Cells were harvested by centrifugation and resuspended in fresh medium. The inoculum was prepared by adjusting the cell suspension to a predetermined optical density (OD) of 0.2 at 600 nm.

For direct contact inhibition, three samples per group were produced with 3 mm (±0.1 mm) in diameter and 1.0 mm (±0.1 mm) thick. The samples were sterilized in hydrogen peroxide plasma. Using a 96-well flat-bottom plate, each specimen was placed on a well containing 300 µL BHI broth (HiMedia Laboratories Pvt. Ltd, Mumbai, India). Then, each well was inoculated with 20 µL of the *E. faecalis* suspension. The negative control consisted of three sets of wells containing uninoculated fresh medium (300 µL). 

Immediately after the placement of inoculums and after 24 h, 90 µL of each well content was diluted in saline to 10^−8^. The 10^−1^, 10^−3^, 10^−6^, and 10^−8^ dilutions were plated in BHI Agar (HiMedia Laboratories), using 25 µL aliquots of each dilution in duplicate. Plates were incubated at 37 °C under anaerobic conditions. After 24 h, the colonies were counted visually, scaled by dilution factors, and then expressed as colony-forming units (CFUs) per milliliters. The groups were statistically compared to each other. The experiment was carried out under aseptic conditions.

### 2.10. Statistical Analysis

Data of radiopacity, flow, film thickness, water sorption, and solubility were analyzed using one-way ANOVA and Tukey. The antibacterial analysis was analyzed via two-way ANOVA (concentration of ZnO-NN and time) and the Student Newman Keuls test. Repeated-measures ANOVA and Tukey analyzed the DC data. All analyses were performed at 0.05 level of significance.

## 3. Results

**Figure 2:**
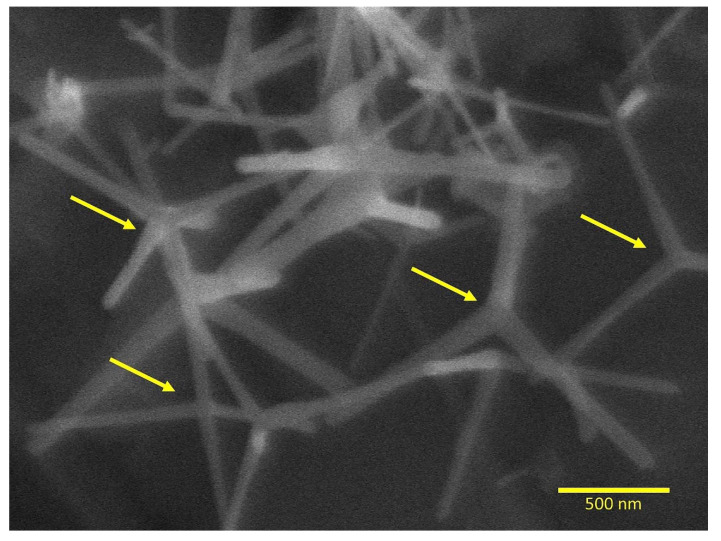
Scanning electron microscopy of ZnO-NN showing the nanostructure after the thermal evaporation process (magnification of 40,000×). The arrows indicate the nucleation core that originates the extensions with distinct directions within three-dimensional space.

Figure 2 shows the SEM images that revealed sharply tapered needle-shaped zinc oxide arms radiating from the center in dense distribution. The mean diameter of the ZnO needles was approximately 40 nm. The surface area by the Brunauer–Emmett–Teller (BET) method showed 16 m²/g for the ZnO-NN.

The flow and film thickness values are displayed in Table 2. The flow of the experimental dental sealer without ZnO-NN addition was unable to measure due to the lack of filler addition, high flowability, so it exceeded the plate limits. The flow of the groups containing ZnO-NN ranged from 17.2 (±2.1) mm for 40 wt.% of ZnO-NN to 18.6 (±1.0) mm for 20 wt.% of ZnO-NN, without statistical difference among groups (*p* > 0.05). The film thickness ranged from 26.7 (±5.0) mm for 0 wt.% to 56.7 (±5.8) mm for 40 wt.% of ZnO-NN. The groups containing ZnO-NN showed higher film thickness in comparison to 0 wt.% (*p* < 0.05) without statistical difference among the three concentrations of ZnO-NN (*p* > 0.5).

Table 2 also displays the results of water sorption and solubility analyses. There was no statistically significant difference among groups in both tests (*p* > 0.05). The radiopacity of the dental sealers ranged from 66.8 (±5.3) pixels to 0 wt.% to 123.2 (±9.0) pixels to 40 wt.% of ZnO-NN. By increasing the concentration of ZnO-NN addition, the higher the radiopacity, with no differences comparing 30 and 40 wt.% of ZnO-NN (*p* > 0.05). The group containing 40 wt.% of ZnO-NN showed no difference from the 1 mm of aluminum (*p* > 0.05).

Table 3 displays the DC of all groups immediately, seven, and 14 days after the manipulation and photoactivation of the dental sealers. Shortly after the photoactivation, the group containing 20 wt.% of ZnO-NN showed no difference in comparison to the group without ZnO-NN addition (*p* > 0.05). Over time, the group containing 40 wt.% of ZnO-NN increased its DC from 2.4 (±1.2) % to 25.9 (±3.4) % after 14 days of storage (*p* < 0.05).

**Figure 3:**
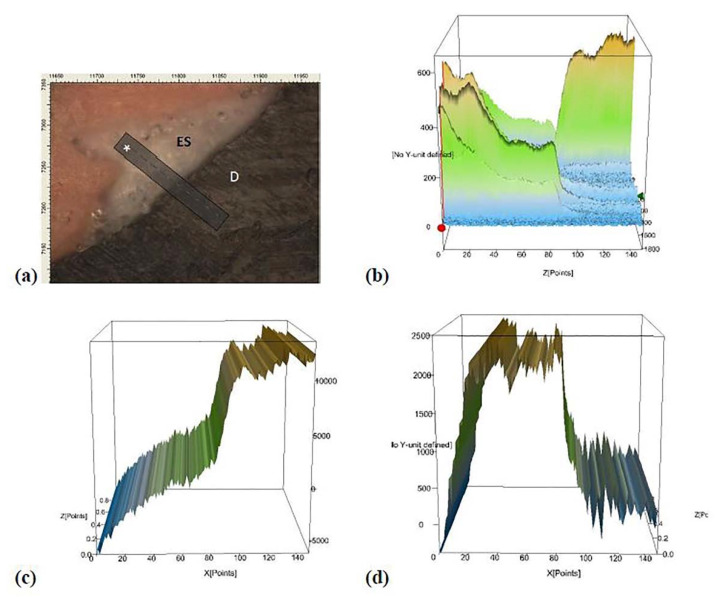
Representative micro-Raman analysis at the dentin-sealer interface. Image (**a**) represents the interface dentin-sealer, where ES states for experimental sealer, D states for dentine, and * is the analyzed line at the interface. Image (**b**) represents the total interface area. Image (**c**) displays the graph when there is integration in micro-Raman analysis for the hydroxyapatite (HAp) using the peak at 910 cm^−1^. Image (**d**) shows the graph when there is integration in the micro-Raman study for ZnO using the peak at 582 cm^−1^. Note that from the sealer analysis into dentin, there is a decrease in the ZnO peak and an increase in the phosphate peak contained in hydroxyapatite (HAp). There is a site in the center of the interface (image **b**) where there is still a high peak of phosphate and the maintenance of the ZnO peak. The overlap of graphs (**c**,**d**) shows the presence of ZnO and phosphate in the same site, suggesting that the ZnO was able to penetrate the dentinal tubules.

Figure 3 shows the micro-Raman results from the analysis of the dental- sealer interface in the root canal. Along with the interface, from the sealer to the dentin, there is a decreased high of ZnO peak and an increase in the peak related to phosphate. The existence of an area in the interface with peaks from ZnO and phosphate suggests that ZnO was in close contact with dentin and may have penetrated the dentinal tubules.

**Figure 4:**
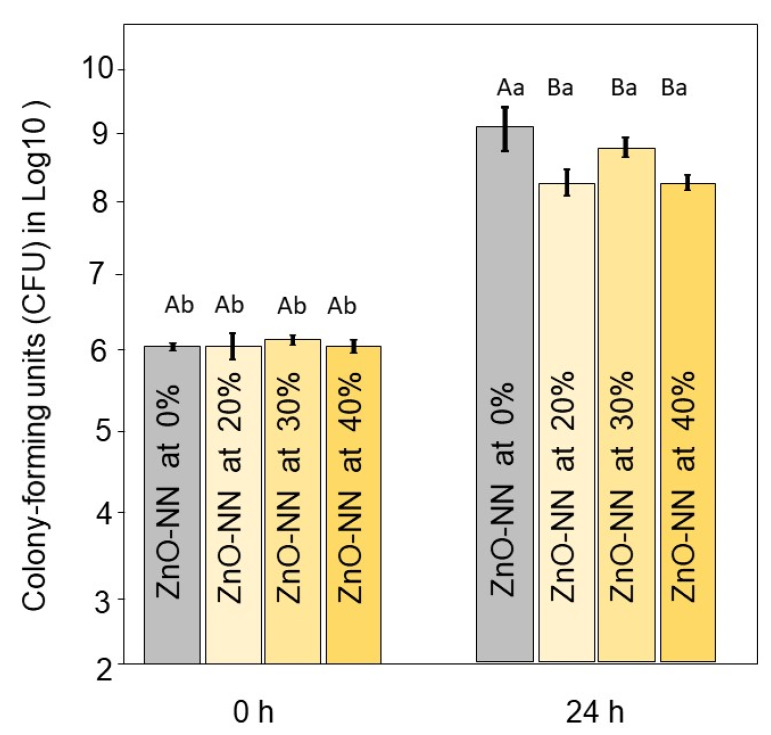
Results of antibacterial analysis of the resin sealers. Mean and standard deviation values of colony-forming units (CFU) in log_10_ at 0 and 24 h of the experimental dental sealers against *E. faecalis*. Different capital letters indicate significant differences among groups within the same time (*p* < 0.05). Different small letters indicate significant differences within the same group at different times (*p* < 0.05).

Figure 4 displays the results of the antibacterial analysis. Immediately after the inoculum, there were no differences among groups (*p* > 0.05). After 24 h of contact with the sealers, the groups with ZnO-NN addition showed a reduced number of bacterial viability when compared to the group without ZnO-NN (*p* < 0.05). At this period, the results ranged from 9.2 (±0.0) log_10_CFU for 0 % of ZnO-NN to 8.3 (±0.2) log_10_CFU for the group with 20 wt.% of ZnO-NN (*p* < 0.05). The groups with 20 to 40 wt.% of ZnO-NN showed no differences among them (*p* > 0.05).

## 4. Discussion

In the present study, needle-like zinc oxide nanostructures (ZnO-NN) were explored as inorganic fillers to improve dental resin sealers. ZnO-NN was successfully synthesized and incorporated in an experimental methacrylate-based root canal sealer. ZnO-NN addition to the sealer increased its radiopacity, maintaining reliable film thickness, flow, pH, and proving activity against *E. faecalis*.

The success of endodontic therapy depends on a complete root canal filling to promote adequate sealing [22]. The sealer should present sufficiently flow to penetrate the irregularities and ramifications of the root canal system to encourage a complete obliteration. The filler size can influence flow and film thickness [33]. The reduction of particle size to nano-range increases the surface area, e.g., 16 m^2^/g of surface area in this study, decreasing the volume fraction that could be added to the polymeric matrix. In the present study, nanosized particles (mean needle diameter of 40 nm) of ZnO were produced and used for sealer formulation. The ZnO-NN addition adjusted the viscosity of the sealer, considering that the group without nanoparticle addition showed a flow that could not be measured (i.e., material flowed beyond the glass plate). One could think that a sealer should present a low viscosity; however, sealers with a high flow could overflow beyond the apex leading to biocompatibility concerns. The ZnO-NN addition improved the handling of the material to be used as an endodontic sealer.

Despite better handling, the materials proposed with ZnO-NN showed flow above 17 mm even with the addition of 40 wt.%, which is following the requirements of ISO 6876:2012 [27]. The film thickness increased with the addition of ZnO-NN (Table 2). However, there was no difference from 20 to 40 wt.% of ZnO-NN (*p* > 0.05). The values found for flow and film thickness were similar to the ones established at ISO 6876:2012 [27] (more than 17 mm and less than 50 µm, respectively). The materials here proposed are probably prone to achieve the apical foramen of dental roots and penetrate under challenging sites in root dentin such as accessory canals, supporting the endodontic treatments.

The wettability of dentin may interfere in the polymeric network formation leading to a resin more prone to long term degradation. It is well known that methacrylate-based materials can present hydrolytic degradation over time in the oral environment [34]. The water sorption and solubility of the polymer could lead to a variety of chemical and physical processes, resulting in harmful effects on the structure and function of dental polymers. These materials can suffer a swelling process [35], and, after swelling, the leaching of unreacted monomers may occur, affecting their stability [36]. Therefore, the experimental materials must not show higher sorption and solubility when in contact with wet environments. In the current study, water sorption and solubility values showed no statistical difference among groups. Lack of solubility has also been stated as an ideal characteristic of root canal filling materials [22] to prevent inflammatory processes in the apical region [23]. 

Low water sorption and solubility can be achieved with the assistance of a suitable DC of carbon-carbon double bonds into carbon-carbon single bonds [37]. A high DC may lead to low solubility, decreasing the amount of unreacted monomer into the polymeric matrix. Moreover, the increased DC is related to improved mechanical properties [38]. The groups with 20 and 30 wt.% of ZnO-NN showed higher DC than 40 wt.%. The reason for that is probably related to the decrease of light energy available within the polymer because it depends on the refractive index of the filler incorporated and the final opacity of the resin-filler composition [39,40]. However, this group with higher ZnO concentration had increased DC overtime after being stored at 37 °C due to the dual-cure system added to the sealers. Overall, all materials presented reliable DC, similar to commercial sealers [41].

Another relevant aspect for sealers is the achievement of radiopacity to make possible the differentiation of the material from the adjacent anatomical structures [30]. ISO 6876:2012 establishes 3 mm of aluminum as the desired radiopacity value for materials to be used as endodontic sealers. The addition of ZnO-NN in all concentrations did not achieve this value. Although the experimental sealers did not meet the ISO requirements, 20 wt.% addition showed higher values than the control group (*p* < 0.05). Moreover, with 40 wt.% of ZnO-NN, the sealers achieved radiopacity compatible with 1 mm of aluminum. Therefore, further modification in the sealer’s composition via the incorporation of radiopacifying agents, i.e., bismuth oxide or ytterbium trifluoride [42], could be performed to surpass their clinical applicability.

The micro-Raman spectra of the sealer/dentin interface suggested the penetration of ZnO-NN into the root canal dentin. The presence of inorganic filler into the dentinal tubules may improve the stability of dentin-methacrylate material interface [43]. Besides, ZnO-NN infiltration into dentin could assist in decreasing collagen degradation by reducing matrix metalloproteinases expression [44] and provide bioactivity for dentin tissue [10].

It is already well established that even after the root canal preparation for endodontic treatment with complete irrigation and shaping, there are still microorganisms on the root canal walls [45]. The improvement of the biological properties of root canal sealers has been studied to overcome this issue [46,47]. The dentinal tubule infection may lead to recurrent disease, determining endodontic retreatment [48]. Here, all concentrations of ZnO-NN addition (20, 30, or 40 wt.%) affected the *E. faecalis* growth assessed by direct contact inhibition.

In the literature, the mechanisms of antibacterial activity of nanostructured ZnO are not well understood, although some statements were proposed, such as the generation of hydrogen peroxide could be the main factor of antibacterial activity [49]. Tiwari’s group have proposed a mechanism of action of ZnO involving the production of reactive oxygen species, which elevates membrane lipid peroxidation that causes membrane leakage of reducing sugars, proteins, DNA, and reduces cell viability [50]. There is also sequential oxidation-reduction reactions, which occur on the ZnO particle surface to produce reactive species such as hydrogen peroxide (H_2_O_2_) and hydroxyl radicals. The reactive oxygen species (ROS) might trigger membrane lipid peroxidation and cause an antibacterial effect.

Some microorganisms escape oxidative stress by living in anaerobic microhabitats; all others must deal with the consequences of intracellular O_2_ [51]. However, almost all of these microorganisms suffer poor growth, elevated mutagenesis, or even death when they are exposed to O_2_ levels that exceed those of their native habitats [52]. *E. faecalis* represents an important clinical problem due to virulence factors such as the regulation of the oxidative stress response by transcriptional regulation by estrogen receptors [53]. A number of antioxidative enzymes have been identified in *E. faecalis* confer protection against hydrogen peroxide, hydroxyl radicals, and superoxide. The defense against other reactive oxygen or nitrogen species is indirect or nonenzymatic.

The use of sealer exhibiting antibacterial properties may be useful to decrease or avoid the growth of the remaining microorganisms in the endodontic system [54]. *E. faecalis* is a recognized pathogen in post-treatment endodontic infections, and it is considered the most resistant species against chemomechanical preparation [55]. Considering the demonstrated potential antimicrobial effect of ZnO-NN and the size-dependent characteristic of this effect, these small particles may contribute to dentin tubules’ disinfection. Usually, the endodontic proposed materials are tested against *E. faecalis* due to the reasons described above. Here, we have estimated the antibacterial activity of sealers containing ZnO-NN using a relevant single-species model. The clinical predictive value of in vitro tests is currently often limited. In this study, even though we observed statistical difference in comparison to 0 wt.% of ZnO-NN, there was no substantial log reduction. The reason for that may be because the test was conducted against planktonic bacteria. Therefore, the leaching of more ZnO-NN to the broth containing *E. faecalis* would be necessary to increase the differences. After 24 h, the material was probably stable enough not to provide a high quantity of free Zn+.

For this reason, further studies could be carried out to understand the effects of the sealers containing ZnO-NN against multi-species biofilm over time. These investigations can be a promising next step to translate the experimental dental sealers containing ZnO-NN into clinical settings.

## 5. Conclusions

In this work, we explored the applicability of ZnO-NN incorporated in an experimental methacrylate-based root canal sealer to impart improved properties. Overall, the incorporation of ZnO-NN provided an antibacterial effect without a significant detrimental impact on the physical and chemical functionality of the material. The use of ZnO-NN as inorganic filler is a potential application within dental materials intended for root canal treatment.

## Figures and Tables

**Table 1:** Description of the components and primary role of each substance used to formulate the base resin of dental sealer formulations.

Chemical Component Description	Abbreviation	Manufacturer	Role of each Substance	Content
Urethane dimethacrylate	UDMA	Sigma-Aldrich, St Louis, MO, USA	Co-monomeric blend composition	70.00 wt.%
Glycerol-1,3-dimethacrylate	GDMA	Sigma-Aldrich, St Louis, MO, USA	Co-monomeric blend composition	15.00 wt.%
Ethoxylated bisphenol A glycol dimethacrylate	BISEMA	Sigma-Aldrich, St Louis, MO, USA	Co-monomeric blend composition	15.00 wt.%
Camphorquinone	CQ	Sigma-Aldrich, St Louis, MO, USA	Photo-initiator: excitation via photoactivation process and reaction with N, N-dihydroxy ethyl-para–toluidine.	1 mol %
N, N-dihydroxy ethyl-para–toluidine	DHEPT	Sigma-Aldrich, St Louis, MO, USA	Co-initiator with CQ and activator with BP: initiation process of the polymerization reaction	1 mol %
Benzoyl-peroxide	BP	Sigma-Aldrich, St Louis, MO, USA	Initiator: initiation process of polymerization reaction with N, N-dihydroxy ethyl-para–toluidine	1 mol %
Nanoneedle structured zinc oxide	ZnO-NN	Self-synthetized	Inorganic filler: improves mechanical properties, reduces hydrolytic degradation, increases viscosity, increases radiopacity	*

* ZnO-NN was added at 0, 20, 30, or 40 wt.% over the total quantity (100 %) of the base resin (monomers and photoinitiator system) of the dental sealer, totalizing four experimental groups.

**Table 2:** Mean and standard deviation (within bracket) values of flow, film thickness, water sorption, and solubility, pH, and radiopacity of the dental sealers.

Groups	Flow (mm)	Film Thickness (mm)	Water Sorption (µm/mm_3_)	Solubility (µm/mm_3_)	Radiopacity (pixels)
0%	*	26.7 (± 5.0) ^B^	39.4 (± 5.6) ^A^	−4.2 (± 0.1) ^A^	66.8 (± 5.3) ^C^
20%	19.8 (± 1.0) ^A^	54.0 (± 12.2) ^A^	32.3 (± 1.7) ^A^	−3.0 (± 1.4) ^A^	103.6 (± 6.9) ^B^
30%	18.6 (± 1.0) ^A^	48.0 (± 7.2) ^A^	35.2 (± 3.8) ^A^	−1.7 (± 1.5) ^A^	110.6 (± 7.9) ^A,B^
40%	17.2 (± 2.0) ^A^	56.7 (± 5.8) ^A^	36.5 (± 6.5) ^A^	0.3 (± 6.1) ^A^	123.2 (± 9.0) ^A,#^

* Flow of experimental sealer without ZnO-NN addition was unable to measure. # The group with 40 wt.% of ZnO-NN showed no statistical difference for 1 mm of aluminum (*p* > 0.05). Different capital letters indicate statistically significant differences in the same column (*p* < 0.05).

**Table 3:** Mean and standard deviation (within bracket) values of degree of conversion (%) of the dental sealers immediately, seven, and 14 days after the photoactivation.

Groups	Immediate	7 Days	14 Days
**0%**	49.9 (± 0.4) ^A,a^	48.1 (± 1.3) ^A,a^	48.1 (± 0.4) ^A,a^
**20%**	46.2 (± 1.5) ^A,B,a^	38.1 (± 2.8) ^B,b^	32.5 (± 2.3) ^B,b^
**30%**	43.0 (± 2.4) ^B,a^	30.0 (± 3.0) ^C,b^	31.5 (± 2.8) ^B,b^
**40%**	2.4 (± 1.2) ^C,b^	26.7 (± 4.6) ^D,a^	25.9 (± 3.4) ^C,a^

Different capital letters indicate statistically significant differences in the same column (*p* < 0.05). Different small letters indicate significant differences in the same line (*p* < 0.05).
